# Impact of extensive antibiotic treatment on faecal carriage of antibiotic-resistant enterobacteria in children in a low resistance prevalence setting

**DOI:** 10.1371/journal.pone.0187618

**Published:** 2017-11-07

**Authors:** Per Kristian Knudsen, Petter Brandtzaeg, E. Arne Høiby, Jon Bohlin, Ørjan Samuelsen, Martin Steinbakk, Tore G. Abrahamsen, Fredrik Müller, Karianne Wiger Gammelsrud

**Affiliations:** 1 Department of Paediatric Medicine, Division of Paediatric and Adolescent Medicine, Oslo University Hospital, Oslo, Norway; 2 Institute of Clinical Medicine, Faculty of Medicine, University of Oslo, Oslo, Norway; 3 Norwegian Institute of Public Health, Oslo, Norway; 4 Department of Methodology Research and Analysis, Domain of Infection Control and Environmental Health, Norwegian Institute of Public Health, Oslo, Norway; 5 Norwegian National Advisory Unit on Detection of Antimicrobial Resistance, Department of Microbiology and Infection Control, University Hospital of North Norway, Tromsø, Norway; 6 Department of Pharmacy, Faculty of Health Sciences, UiT The Arctic University of Norway, Tromsø, Norway; 7 Department of Antibiotic Resistance and Infection Prevention, Domain of Infection Control and Environmental Health, Norwegian Institute of Public Health, Oslo, Norway; 8 Department of Microbiology, Division of Laboratory Medicine, Oslo University Hospital, Oslo, Norway; Hospital for Sick Children, CANADA

## Abstract

We prospectively studied the consequences of extensive antibiotic treatment on faecal carriage of antibiotic-resistant enterobacteria in a cohort of children with cystic fibrosis (CF) and a cohort of children with cancer compared to healthy children with no or low antibiotic exposure. The study was conducted in Norway in a low resistance prevalence setting. Sixty longitudinally collected faecal samples from children with CF (*n* = 32), 88 samples from children with cancer (*n* = 45) and 127 samples from healthy children (*n* = 70) were examined. A direct MIC-gradient strip method was used to detect resistant *Enterobacteriaceae* by applying Etest strips directly onto agar-plates swabbed with faecal samples. Whole genome sequencing (WGS) data were analysed to identify resistance mechanisms in 28 multidrug-resistant *Escherichia coli* isolates. The prevalence of resistance to third-generation cephalosporins, gentamicin and ciprofloxacin was low in all the study groups. At inclusion the prevalence of ampicillin-resistant *E*. *coli* and trimethoprim-sulfamethoxazole-resistant *E*. *coli* in the CF group compared to healthy controls was 58.6% vs. 28.4% (p = 0.005) and 48.3% vs. 14.9% (p = 0.001), respectively, with a similar prevalence at the end of the study. The prevalence of resistant enterobacteria was not significantly different in the children with cancer compared to the healthy children, not even at the end of the study when the children with cancer had been treated with repeated courses of broad-spectrum antibiotics. Children with cancer were mainly treated with intravenous antibiotics, while the CF group mainly received peroral treatment. Our observations indicate that the mode of administration of antibiotics and the general level of antimicrobial resistance in the community may have an impact on emergence of resistance in intestinal enterobacteria during antibiotic treatment. The WGS analyses detected acquired resistance genes and/or chromosomal mutations that explained the observed phenotypic resistance in all 28 multidrug-resistant *E*. *coli* isolates examined.

## Introduction

The worldwide increasing prevalence of antimicrobial resistance with decreased access to effective antimicrobials has become one of the biggest health care challenges of our time [1, 2]. Use and overuse of antibiotics in humans is one of the main drivers of antibiotic resistance although the relationship between human antimicrobial usage and resistance is complex [3, 4]. Several studies have documented an association between antibiotic use in humans and the development of resistance both at a population level and in individuals [5–7].

During antibiotic treatment both pathogens and commensals are exposed to antibiotics. Several studies have shown effects of antibiotic treatment on the human gut flora [8–10], including selection of antibiotic-resistant enterobacteria [11]. Resistant endogenous enterobacteria may cause infections that are difficult to treat, and the gut microbiota may serve as a reservoir for antibiotic resistance genes [12, 13]. These resistance genes may spread between bacterial strains and species within the microbiome [14–16], between individuals, and to the environment [17, 18].

The studies on effects of antibiotic treatment on intestinal bacteria have mainly revealed an increased level of resistance during and after single courses of treatment [8, 11]. The studies show that after antibiotic treatment, the susceptibility either returns to baseline levels shortly after the cessation of therapy [11, 19], or there is a prolonged effect even after single courses [20–22]. Given the fact that systematic, longitudinal studies of the consequences of long-time extensive antibiotic exposure in infants and children are lacking, we have investigated the faecal flora in children with cystic fibrosis (CF) and cancer, two patient groups known to receive much antibiotic treatment. Healthy children were included as a control group. The prevalence of antibiotic resistance was low in Norway during the study period [23].

CF is an inherited disease caused by mutations in the cystic fibrosis transmembrane conductance regulator (CFTR) gene leading to altered ion and water transport across apical cell membranes in exocrine glands [24]. Viscous airway secretions and reduced mucociliary clearance lead to frequent and chronic lung infections. Consequently, CF patients are repeatedly treated with antibiotics from early childhood [25, 26]. Children with cancer are often treated with chemotherapy that causes immunosuppression including neutropenia. This entails an increased risk of severe invasive infections requiring repeated courses with broad-spectrum antibiotics.

The primary aim of this study was to investigate whether the prevalence of faecal carriage of antibiotic-resistant enterobacteria is higher in children with CF or cancer compared to healthy children with low antibiotic exposure in Norway. The secondary aim was to explore the genetic determinants conferring antibiotic resistance in intestinal enterobacteria isolated from these children.

## Materials and methods

### Study design, participants and clinical information

Serial faecal samples were collected from children with CF (*n* = 32, 2004–2007) and children with cancer (*n* = 45, 1999–2000 and 2003–2005) treated at Oslo University Hospital, Oslo, Norway and from 70 healthy children in day-care centres and schools in Oslo (2000–2001 and 2006–2008). Age and gender distributions are shown in Table 1. CF patients were consecutively included at regular outpatient visits, and the faecal samples were collected regardless of clinical condition or on-going antibiotic treatment. Cancer patients were consecutively included within one week after cancer was diagnosed. The first faecal sample was collected regardless of prior or on-going antibiotic treatment, since such treatment often was initiated prior to or shortly after admission to the hospital.

**Table 1:** Background information and faecal sample information on the children with cystic fibrosis, the children with cancer and the healthy children.

	Cystic fibrosis (n = 32)	Cancer (n = 45)	Healthy controls (n = 70)	p-values	
				CF vs. HC	C vs. HC
Age (year) [median (range) ]	5.9 (0.9–16.3)	4.3 (0.3–14.1)	5.5 (0.5–15.5)	0.91	0.23
Female gender [n (%)]	15 (46.9)	23 (51.1)	38 (54.3)	0.49	0.74
Months between first and last sample [median (range)]	9.1 (4.2–22.1)	8.0 (1.5–16.4)	13.4 (3.0–26.0)	0.02	<0.001
Individuals with two samples^a^ [n (%)]	28 (87.5)	43 (95.6)	57 (81.4)	0.45	0.03

CF, cystic fibrosis; C, cancer; HC, healthy controls.

^a^ One sample obtained from the remaining individuals.

Inclusion criteria were children diagnosed with CF based on clinical symptoms and a positive sweat test and/or two identified CFTR mutations, children with newly diagnosed cancer who received treatment with at least one course of anticancer chemotherapy, and healthy children with no underlying chronic disease. The cancer diagnoses comprised 19 acute lymphoblastic leukaemias, six acute myeloblastic leukaemias, six non-Hodgkin lymphomas, four Wilms tumours, two primitive nevroectodermal tumours, and one each of ependymoma, Ewing sarcoma, germinal cell tumour, Hodgkin lymphoma, opticus glioma, rhabdomyosarcoma, rhabdoid kidney tumour and spindle-celled sarcoma.

For children with CF and cancer, the medical records were reviewed for antibiotic treatment during the study period. In addition, parents in all three study groups were asked about previous antibiotic treatment. Written, informed consent was obtained from the participants’ parents and from the participants themselves when 12 years or older. The study was approved by the Regional Committee for Medical and Health Research Ethics–South East (“REK sør-øst”) (reference number 581-06-03092).

### Detection of resistant *Enterobacteriaceae* in faecal samples

The faecal samples were directly mixed with Carey-Blair transport medium and either sent by mail or personally delivered to the laboratory. The samples were then plated out on lactose agar plates and incubated at 35°C (0–5 days after sampling). Bacterial growth (mainly *Enterobacteriaceae*) was noted, and morphologically different colonies were selected and identified. Both primary growth and the identity of the selected bacteria were recorded for each sample. Furthermore, the pure cultures and the faecal samples, mixed with Greaves`freezing medium, were frozen consecutively at -80°C. For the present study a direct MIC-gradient strip method, recently described by Gammelsrud et al. [27], was used to detect resistant *Enterobacteriaceae* in the faecal samples. In short, the stool sample suspensions were thawed and swabbed directly onto two 14 cm diameter Mueller-Hinton agar plates (Becton Dickinson, Sparks, MD, USA). Six MIC-gradient strips (Etest, bioMeriéux, Marcy L’Étoile, France) were subsequently applied directly onto each plate. The following 12 antibiotics were used; ampicillin, aztreonam, cefotaxime, cefoxitin, ceftazidime, ciprofloxacin, colistin, gentamicin, imipenem, tetracycline, tobramycin and trimethoprim-sulfamethoxazole. All samples were also plated on a lactose agar plate as growth control for comparison with the initial growth prior to the freezing. All plates were incubated for 18–24 h at 35°C in ambient air. Only Gram-negative bacteria were further studied. In the case of growth within the Etest ellipses (due to assumingly more resistant organisms than the dominant bacterial populations), 1–2 colonies of each morphotype were selected from the ellipse area for species identification and further susceptibility testing. To assess the dominant bacterial populations with confluent growth along the Etests, a loop-full of growth next to the strip at the highest MIC value was inoculated onto a lactose agar plate. One colony of each morphotype from this growth was also subsequently selected for species identification and susceptibility testing. Species identification was done by the three-tube fermentation method [28] or by MALDI-TOF MS (Bruker Daltonics, Bremen, Germany). All selected isolates were tested against nine antibiotics (ampicillin, cefotaxime, ceftazidime, ciprofloxacin, gentamicin, imipenem, meropenem, tetracycline and trimethoprim-sulfamethoxazole) by disk diffusion according to EUCAST (v 6.0 2016, www.eucast.org). For isolates classified as intermediately susceptible, the final susceptibility category was confirmed using Etest.

### Whole genome sequencing (WGS) and assembling

To explore genetic determinants responsible for antibiotic resistance, WGS was applied on 28 *Escherichia coli* isolates. These were the *E*. *coli* isolates from the CF group (n = 10), the cancer group (n = 5) and the control group (n = 2) displaying phenotypic resistance to one or more of the following antibiotics: cefotaxime, ceftazidime, ciprofloxacin and gentamicin, and all other *E*. *coli* isolates from the CF group (n = 11) simultaneously resistant to antibiotics from at least three antimicrobial categories [29].

Genomic DNA was extracted using MagNa Pure 96 (Roche Diagnostics, Mannheim, Germany) according to the manufacturer's instructions. DNA concentrations were measured using a Qubit fluorometer (Thermo Fisher Scientific, MA, USA) to determine DNA input for each isolate. Libraries were prepared using KAPA HyperPlus Library Preparation Kit (Kapa Biosystems, MA, USA). WGS was performed on the Illumina MiSeq platform using v 2 reagent kits generating 2x250 bp paired-end reads (Illumina, San Diego, CA, USA). All isolates were quality corrected and assembled using BayesHammer/SPAdes v 3.6.0. [30]. Assembly information about the different isolates can be found in S1 Table.

### Identification of genetic resistance determinants and multilocus sequence typing (MLST) of *E*. *coli*

The assembled genomes were submitted to the web-based ResFinder service v 2.1 (Center for Genomic Epidemiology, DTU, Denmark) to identify acquired resistance genes [31]. Hits were accepted for matches with ≥ 99% nucleotide identity and length of the query sequence covering ≥ 95% of the length of the gene in the database. If acquired resistance genes associated with the phenotypic resistance of the isolate were not found by ResFinder, Python scripts were written to extract *gyrA*, *gyrB*, *parC*, *parE* and *ampC* genes. The quinolone resistance-determining regions (QRDRs) of the *gyrA*, *gyrB*, *parC* and *parE* genes or the promoter and attenuator regions of the *ampC* gene, as appropriate, were analysed to identify chromosomal structural gene mutations by alignment using Seaview v 4.6.1. [32].

MLST of all the sequenced *E*. *coli* isolates was performed from WGS data by the web-based MLST v 1.8 service (Center for Genomic Epidemiology, DTU, Denmark) [33], using two different *E*. *coli* MLST schemes [34, 35].

### Identification of resistance mechanisms in *Enterobacteriaceae* species other than *E*. *coli*

Non-*E*. *coli* isolates with reduced susceptibility to third-generation cephalosporins were investigated for the phenotypic expression of extended-spectrum β-lactamases (ESBLs) and AmpC using Etest combination gradient-strips (bioMeriéux, Marcy L’Étoile, France), combination discs (AmpC confirm ID kit, Rosco Diagnostica, Taastrup, Denmark) and BD ESBL discs (BD Diagnostic Systems, Sparks, USA).

### Statistics

Pearson's chi-square test was used when comparing categorical data in two groups, but Fischer's exact test was used when the overall sample size was <40 and the smallest expected number was <5 in the 2x2 table analysis. Independent samples t-test was used when comparing normally distributed continuous data in two groups (SPSS software, v 22.0). The significance level was set to 5%.

## Results

### Faecal samples and enterobacterial species distribution

The total number of faecal samples collected from each participant differed from one to 15 (median three). From some of the samples there was no growth of Gram-negative enterobacteria despite several attempts to inoculate the faecal specimens on different agars, possibly due to recent or on-going antibiotic exposure. For this study, the first and the last faecal sample that showed growth of *Enterobacteriaceae* from each participant were included. From four children with CF, two children with cancer and 13 healthy controls, we either received only one faecal sample or only one of the submitted samples showed enterobacterial growth. In all, 29 CF patients, 44 cancer patients and 67 healthy controls submitted samples with growth of enterobacteria at the time of inclusion (first sample), whereas 31 CF patients, 44 cancer patients and 60 controls submitted samples with enterobacterial growth at the end of the study period (last sample). Sample information is presented in Table 1.

The observed growth of enterobacteria on the lactose agar after storage of the faecal samples was in high agreement with the primary growth before freezing of the samples.

From each sample, 0–5 phenotypically different isolates of *Enterobacteriaceae* species were detected. The proportions of the faecal samples from the three study groups with growth of different enterobacterial species are shown in Table 2.

**Table 2:** Growth of different *Enterobacteriaceae* species in faecal samples from 32 children with cystic fibrosis, 45 children with cancer and 70 healthy children^a^.

		CF, n/N (%)	Cancer, n/N (%)	Healthy controls, n/N (%)	p-values	
					CF vs. HC	C vs. HC
***E*.*coli***						
	First sample	26/29 (89.7)	41/44 (93.2)	63/67 (94.0)	0.45	0.86
	Last sample	30/31 (96.8)	37/44 (84.1)	58/60 (96.7)	0.98	0.02
***Klebsiella* spp.**						
	First sample	17/29 (58.6)	15/44 (34.1)	14/67 (20.9)	<0.001	0.12
	Last sample	16/31 (51.6)	13/44 (29.5)	9/60 (15.0)	<0.001	0.07
***Enterobacter* spp.**						
	First sample	9/29 (31.0)	10/44 (22.7)	8/67 (11.9)	0.02	0.13
	Last sample	8/31 (25.8)	12/44 (27.3)	10/60 (16.7)	0.30	0.19
***Citrobacter* spp.**						
	First sample	5/29 (17.2)	5/44 (11.4)	11/67 (16.4)	0.92	0.46
	Last sample	8/31 (25.8)	6/44 (13.6)	9/60 (15.0)	0.21	0.85
**Other *Enterobacteriaceae* spp.**^**b**^						
	First sample	9/29 (31.0)	3/44 (6.8)	8/67 (11.9)	0.02	0.38
	Last sample	9/31 (29.0)	6/44 (13.6)	2/60 (3.3)	<0.001	0.05

CF, cystic fibrosis; C, cancer; HC, healthy controls.

^a^ The total number (N) of first samples and last samples differ within each study group because some individuals submitted only one faecal sample with enterobacterial growth, either at the inclusion or at the end of the study period.

^b^ *Hafnia alvei*, *Proteus* spp., *Morganella morganii*, *Yersinia enterocolitica*, *Kluyvera intermedia*

### Antibiotic consumption

#### Children with CF or cancer

**Fig 1:**
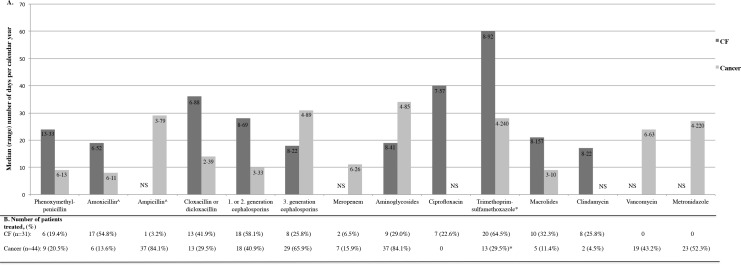
Antibiotic treatment in 31 children with cystic fibrosis (CF) and 44 children with cancer. (A) Median (range, shown inside the columns) number of days per calendar year with antibiotic treatment in the study period among patients who received at least one course of treatment. For some antibiotics, median number of days is not shown (NS) due to no or only one or two patients treated. (B) Number of patients (%) in each patient group treated with at least one course of the antibiotic. For one of the 32 CF patients and for one of the 45 cancer patients included in the study no antibiotic treatment was registered since a faecal sample was provided only at the time of inclusion into the study. ^Amoxicillin was only administered orally and ampicillin was only administered intravenously. *Prophylactic treatment given to cancer patients is not included.

Children in both patient groups received numerous antibiotic courses during the study period, but the treatment pattern differed significantly between these two groups, as shown in Fig 1. For detailed information, see S2 Table. Aminopenicillin was almost exclusively administered intravenously (ampicillin) to the cancer patients, whilst given orally (amoxicillin) to the CF patients. Seventy percent of the cancer patients received prophylaxis against *Pneumocystis jirovecii* with trimethoprim-sulfamethoxazole 2–3 days per week. Prophylactic treatment was not given to any CF patient. Five of the CF patients were treated with inhaled tobramycin while six received inhaled colistin during the study period. No cancer patient received inhaled antibiotics. Parents of cancer patients reported antibiotic treatment before the cancer diagnosis for 26 of the 45 children (58%). Eight of them were treated within two months prior to inclusion in the study, and 33 cancer patients received antibiotics after study inclusion, but prior to the first faecal sample. This treatment is included in Fig 1 and S2 Table. All the CF patients had received multiple courses of antibiotics prior to the study, but this is not included in Fig 1 and S2 Table.

#### Healthy controls

According to parental information, a total of 37 of the 70 healthy children (53%) had never been treated with antibiotics prior to or during the study period. The remaining 33 children had received mainly a single course or a few courses of peroral phenoxymethylpenicillin, amoxicillin or erythromycin. The antibiotic treatment in each of the healthy children is shown in S3 Table. Nine (13%) of the healthy children had received antibiotic treatment within a year before the first study sample, including four treated during the last two months. Five children (7.1%) received antibiotics during the study period including four less than one year but only one less than two months prior to the last sample.

### Prevalence of antibiotic resistance

Table 3 shows the prevalence of *E*. *coli* and all enterobacterial species combined (including *E*. *coli*) resistant to the antibiotics tested in the two faecal samples from our three study groups. No significant difference between the first and the last sample was observed for any of the tested antibiotics in any of the three groups.

**Table 3:** Prevalence of antibiotic-resistant isolates of *Escherichia coli* and all *Enterobacteriaceae* species combined (including *E*. *coli*) in the first and the last faecal sample from children with cystic fibrosis, cancer and healthy controls (one or more resistant isolates per sample)^a^.

		CF, n/N (%)	Cancer, n/N (%)	Healthy controls, n/N (%)	p-values	
					CF vs. HC	C vs. HC
**Ampicillin**						
*E*. *coli*						
	First sample	17/29 (58.6)	12/44 (27.3)	19/67 (28.4)	0.005	0.90
	Last sample	19/31 (61.3)	17/44 (38.6)	20/60 (33.3)	0.01	0.58
All *Enterobacteriaceae* spp.						
	First sample	24/29 (82.8)	27/44 (61.4)	36/67 (53.7)	0.007	0.43
	Last sample	28/31 (90.3)	29/44 (65.9)	34/60 (56.7)	0.001	0.34
**Trimethoprim-sulfamethoxazole**						
*E*. *coli*						
	First sample	14/29 (48.3)	8/44 (18.2)	10/67 (14.9)	0.001	0.65
	Last sample	14/31 (45.2)	12/44 (27.3)	11/60 (18.3)	0.007	0.28
All *Enterobacteriaceae* spp.						
	First sample	15/29 (51.7)	9/44 (20.5)	10/67 (14.9)	<0.001	0.45
	Last sample	18/31 (58.1)	14/44 (31.8)	11/60 (18.3)	<0.001	0.11
**Tetracycline**						
*E*. *coli*						
	First sample	13/29 (44.8)	9/44 (20.5)	15/67 (22.4)	0.03	0.81
	Last sample	9/31 (29.0)	9/44 (20.5)	16/60 (26.7)	0.81	0.46
All *Enterobacteriaceae* spp.						
	First sample	16/29 (55.2)	12/44 (27.3)	17/67 (25.4)	0.005	0.82
	Last sample	14/31 (45.2)	11/44 (25.0)	18/60 (30.0)	0.07	0.57
**Cefotaxime and/or Ceftazidime**						
*E*.*coli*						
	First sample	1/29 (3.4)	2/44 (4.5)	0/67	0.13	0.08
	Last sample	2/31 (6.5)	1/44 (2.3)	0/60	<0.05	0.24
All *Enterobacteriaceae* spp.						
	First sample	1/29 (3.4)	4/44 (9.1)	3/67 (4.5)	0.82	0.33
	Last sample	2/31 (6.5)	4/44 (9.1)	3/60 (5.0)	0.77	0.41
**Ciprofloxacin**						
*E*.*coli*						
	First sample	2/29 (6.9)	1/44 (2.3)	1/67 (1.5)	0.16	0.76
	Last sample	1/31 (3.2)	1/44 (2.3)	0/60	0.16	0.24
All *Enterobacteriaceae* spp.						
	First sample	2/29 (6.9)	1/44 (2.3)	1/67 (1.5)	0.16	0.76
	Last sample	1/31 (3.2)	1/44 (2.3)	0/60	0.16	0.24
**Gentamicin**						
*E*.*coli*						
	First sample	1/29 (3.4)	1/44 (2.3)	2/67 (3.0)	0.91	0.82
	Last sample	1/31 (3.2)	1/44 (2.3)	0/60	0.16	0.24
All *Enterobacteriaceae* spp.						
	First sample	1/29 (3.4)	1/44 (2.3)	2/67 (3.0)	0.91	0.82
	Last sample	1/31 (3.2)	1/44 (2.3)	0/60	0.16	0.24

CF, cystic fibrosis; C, cancer; HC, healthy controls.

^a^ The total number (N) of first samples and last samples differ within each study group because some individuals submitted only one faecal sample with enterobacterial growth, either at the inclusion or at the end of the study period.

#### Children with cancer

The prevalence of *E*. *coli* and all *Enterobacteriaceae* species combined, resistant to the antibiotics tested was not significantly different in the children with cancer compared to the healthy children in any of the two samples (Table 3).

#### Children with CF

The prevalence of ampicillin-resistant and trimethoprim-sulfamethoxazole-resistant *E*. *coli* and *Enterobacteriaceae* species combined was significantly higher in the CF group compared to the healthy children (Table 3).

Within the CF group the relative risk (RR) of harbouring trimethoprim-sulfamethoxazole-resistant *Enterobacteriaceae* in the last faecal sample was 1.83 (95%CI 1.11–3.02) in those treated with trimethoprim-sulfamethoxazole during the last 60 days compared to those not treated. Other statistically significant correlations between antibiotic exposure and antibiotic resistance were not found for any antibiotics in any of the two patient groups.

#### Healthy controls

Enterobacteria with resistance to the same type of antibiotic that the children had been treated with, were detected in only three of the 13 samples that were collected from the healthy children less than one year after the antibiotic treatment. These were ampicillin-resistant *E*. *coli* isolates detected in samples from children that had received amoxicillin.

No carbapenem-resistant *E*. *coli* or other *Enterobacteriaceae* species were found in any of the three study groups.

The prevalence of *E*. *coli* resistant to ampicillin and trimethoprim-sulfamethoxazole was not significantly different in children younger than four years compared to children older than four years in any of the three study groups (Table 4). A shift in ampicillin and trimethoprim-sulfamethoxazole susceptibility in *E*. *coli* between the first and the last sample (resistant isolates detected in the last sample but not in the first sample or vice versa) was observed in children from all the study groups (Table 5).

**Table 4:** Prevalence of ampicillin-resistant and trimethoprim-sulfamethoxazole-resistant *E*. *coli* in faecal samples from two age groups (younger or older than 4 years) of patients with cystic fibrosis, cancer and healthy controls (first study sample).

Antibiotic resistance	Study groups	< 4 years, n/N (%)	> = 4 years, n/N (%)	p-value
**Ampicillin**	Cystic fibrosis	7/12 (58.3)	10/17 (58.8)	1.0
	Cancer	4/21 (19.0)	8/23 (34.8)	0.24
	Healthy controls	6/20 (30.0)	13/47 (27.7)	0.85
**Trimethoprim-sulfamethoxazole**	Cystic fibrosis	6/12 (50.0)	8/17 (47.1)	0.88
	Cancer	2/21 (9.5)	6/23 (26.1)	0.16
	Healthy controls	2/20 (10.0)	8/47 (17.0)	0.46

**Table 5:** Occurrence of shift in ampicillin and trimethoprim-sulfamethoxazole susceptibility in faecal *Escherichia coli* between the first and the last faecal sample collected from children with cystic fibrosis, cancer and healthy controls.

		No resistant isolates in the first sample, resistant isolates in the last sample, n/N (%)	Resistant isolates in the first sample, no resistant isolates in the last sample, n/N (%)	Same resistance pattern in the first and the last sample, n/N (%)
**Ampicillin- resistance**	Cystic fibrosis	7/28 (25.0)	6/28 (21.4)	15/28 (53.6)
	Cancer	8/43 (18.6)	2/43 (4.7)	33/43 (76.7)
	Healthy controls	10/57 (17.5)	7/57 (12.3)	40/57 (70.2)
**Trimethoprim-sulfamethoxazole- resistance**	Cystic fibrosis	3/28 (10.7)	5/28 (17.9)	20/28 (71.4)
	Cancer	6/43 (14.0)	2/43 (4.7)	35/43 (81.4)
	Healthy controls	8/57 (14.0)	6/57 (10.5)	43/57 (75.4)

### Antibiotic resistance mechanisms

To investigate the mechanisms behind the observed resistance in *E*. *coli* isolates, 28 isolates were genome sequenced and WGS data were analysed for acquired resistance genes and specific chromosomal mutations (Table 6, S4 Table). Resistance to ampicillin and third-generation cephalosporins was mainly due to the presence of narrow-spectrum β-lactamases (*bla*_TEM-1_), ESBLs (*bla*_CTX-M-1_ and *bla*_SHV-2_) or plasmid-mediated AmpC (*bla*_CMY-7_) [36]. In two isolates resistant to third-generation cephalosporins, but devoid of acquired ESBLs, previously described *bla*_AmpC_ promoter mutations (-42:C->T and -18:G->A) [37] were identified. One or two *dfrA* genes in combination with one or two *sul* genes were found in all isolates resistant to trimethoprim-sulfamethoxazole [38] and at least one of the tetracycline efflux genes *tet(A)*, *tet(B)* or *tet(D)* were found in all tetracycline-resistant isolates [39]. Two *gyrA* mutations (Ser-83-Leu and Asp-87-Asn) and at least one *parC* mutation (Ser-80-Ile or Glu-84-Val) were detected in the QRDRs in all seven ciprofloxacin-resistant isolates [40]. No plasmid-mediated resistance genes associated with quinolone resistance were detected. Variants of *aac* genes or the *aadB* (= ANT(2”)-1a) gene were detected in the gentamicin-resistant isolates [41].

**Table 6:** Genotypic resistance mechanisms identified in 28 resistant *Escherichia coli* isolates from faecal samples from children with cystic fibrosis (21 isolates), cancer (5 isolates) and healthy children (2 isolates).

Phenotypic resistance	Number of isolates	Acquired resistance genes	Chromosomal mutations
Ampicillin and third-generation cephalosporins	28 ampicillin-resistant (21 CF, 5 C, 2 HC), 10 third-generation cephalosporin-resistant (6 CF, 4 C)	*bla*_TEM-1_ (21 isolates), *bla*_CTX-M-1_ (5 isolates)^a^, *bla*_SHV-2_ (1 isolate), *bla*_CMY-7_ (2 isolates)^b^	*ampC* promoter mutations: -42: C→T and -18:G→A (2 isolates)
Trimethoprim-sulfamethoxazole	22 (18 CF, 3 C, 1 HC)	*dfrA1* (5 isolates), *dfrA7* (3 isolates), *dfrA12* (1 isolate), *dfrA14* (3 isolates), *dfrA17* (12 isolates)^c^ + *sul1* (16 isolates), *sul2* (19 isolates)^d^	
Tetracycline	21 (17 CF, 2 C, 2 HC)	*tet(A)* (14 isolates), *tet(B)* (7 isolates), *tet(D)* (2 isolates)^e^	
Gentamicin	7 (2 CF, 3 C, 2 HC)	*aac(3)-IIa* (1 isolate), *aac(3)-IId* (4 isolates)^b^, *aac(3)-IVa* (1 isolate), *aadB* (1 isolate)	
Ciprofloxacin	7 (3 CF, 3 C, 1 HC)		2 *gyrA* mutations: Ser-83-Leu, Asp-87-Asn (7 isolates)^b^ + 1 *parC* mutation: Ser-80-Ile (6 isolates)^b^ or 2 *parC* mutations: Ser-80-Ile, Glu-84-Val (1 isolate)

CF, cystic fibrosis; C, cancer; HC, healthy children

^a^ The five isolates harbouring the *bla*_CTX-M-1_ gene were isolated from one CF patient and represented two different *E*. *coli* sequence types (STs) from the first faecal sample and the same two STs plus an additional ST from the last sample collected.

^b^ Two of the isolates from one cancer patient represented the same *E*. *coli* ST but with different tetracycline susceptibility patterns.

^c^ Two different *dfrA* genes were found in two of the isolates.

^d^ Both the *sul1* and the *sul2* gene were found in 13 of the isolates.

^e^ Two different *tet* genes were found in two of the isolates.

Detailed information about each of the isolates can be found in S4 Table.

Phenotypic analysis of the identified *Enterobacteriaceae* species other than *E*. *coli* with reduced susceptibility to third-generation cephalosporins showed that all were negative for the presence of ESBLs. Details can be found in S5 Table.

The overall prevalence (in the first and/or the last faecal sample) of *Enterobacteriaceae* carrying acquired ESBLs was 1/32 (3.1%) in the CF group and 2/45 (4.4%) in the cancer group. No ESBL-producing enterobacteria were detected in the healthy control group.

### *E*. *coli* MLST analyses

MLST data from the 28 sequenced *E*. *coli* isolates are shown in S4 Table. Isolates with identical sequence type (ST69) were detected in samples from two different individuals (both with CF) on only one occasion. Five isolates carrying *bla*_CTX-M-1_ isolated from one single CF patient comprised three sequence types (STs); two different STs (ST1640 and ST6331) were detected in the first sample, and the same two STs in addition to a third ST (ST2144) were detected in the last sample.

## Discussion

In this study we compared the prevalence of faecal carriage of resistant enterobacteria in two different patient groups of children with high antibiotic exposure to a group of healthy children with low or no known previous antibiotic exposure. We found the prevalence of resistance against ampicillin and trimethoprim-sulfamethoxazole to be significantly higher in the children with CF compared to the healthy children (Table 3). In the last faecal samples collected at the end of the study, the prevalence of ampicillin-resistant *E*. *coli* was 61.3% and the prevalence of trimethoprim-sulfamethoxazole-resistant *E*. *coli* was 45.2% in the CF group, significantly higher than in the group of healthy children (33.3% and 18.3%, respectively). Surprisingly, the prevalence of antibiotic-resistant enterobacteria was not significantly different in the children with cancer as compared to the healthy children, not even at the end of the study period when the children with cancer had been treated with repeated courses of broad-spectrum antibiotics.

The level of enterobacterial resistance was low in Norway in the period the study samples were collected [23]. The prevalence of resistant faecal *E*. *coli* in the group of healthy children in our study was similar to the level of resistance in clinical *E*. *coli* isolates from urinary tract infections reported from the Norwegian Surveillance System for Antimicrobial Drug Resistance [23]. The prevalence of faecal *E*. *coli* resistant to ampicillin, trimethoprim-sulfamethoxazole, tetracycline, ciprofloxacin or third-generation cephalosporins in the control group was also at the same level as the corresponding pooled prevalence of resistant, faecal *E*. *coli* in children in countries within the Organisation for Economic Co-operation and Development (OECD) [22]. Fifty-three percent of the children in the healthy control group had never been treated with antibiotics and the majority of the other children had received only a single or very few courses of phenoxymethylpenicillin, amoxicillin or erythromycin more than one year prior to inclusion in the study, based on the parents reporting of drug use (S3 Table). We detected enterobacteria that were resistant to the same antibiotic that the child had been treated with in only three of the 13 faecal samples that were collected less than one year after antibiotic exposure. Thus, it seems that this previous, often Gram-positive spectrum, antibiotic treatment had little or no impact on the overall level of antibiotic resistance in the group of healthy children, justifying the use of these children as a control group in our study. We found no significant difference in the prevalence of ampicillin- and trimethoprim-sulfamethoxazole-resistant *E*. *coli* when we compared children younger or older than four years of age within any of our three study groups, indicating that resistant faecal *E*. *coli* is established at an early age, in accordance with previous studies [42–44].

In one small study the prevalence of amoxicillin-resistant *Enterobacteriaceae* in faecal samples from two children with CF was higher than in samples from their healthy siblings [45]. Only two other studies have investigated the prevalence of resistant faecal enterobacteria in CF patients, reporting no increase in resistance after treatment with ciprofloxacin and ceftazidime, respectively [46, 47]. Other studies, in other patient groups or healthy volunteers, have reported an increase in resistant bacteria in stools after antibiotic exposure [8, 11, 22, 48]. The significantly higher prevalence of ampicillin- and trimethoprim-sulfamethoxazole-resistant enterobacteria in CF patients in our study is likely due to their large exposure to these antibiotics; 55% of the CF patients were treated with per oral amoxicillin for a median of 19 days per calendar year during the study period, and 65% were treated with trimethoprim-sulfamethoxazole for a median of 60 days. We also found a relative risk (RR) of 1.83 (95%CI 1.11–3.02) for carrying trimethoprim-sulfamethoxazole-resistant enterobacteria in the last faecal sample in CF patients treated with trimethoprim-sulfamethoxazole within the last 60 days as compared to those not treated. Selection of resistant bacteria during antibiotic treatment is the most likely mechanism causing the higher presence of resistant faecal enterobacteria in children with CF, and facilitation of horizontal transfer of resistance genes during antibiotic exposure may also have occurred [15, 49].

Most of our CF patients had received substantial amounts of antibiotics prior to inclusion in the study. This may explain why the prevalence of resistance was at the same level in the first and last sample in the CF group (Table 3). However, a shift in susceptibility patterns between the first and the last sample was noticed for ampicillin-resistant and trimethoprim-sulfamethoxazole-resistant *E*. *coli* in children from all three study groups (Table 5). This reflects the dynamic nature of the faecal flora and clearly underlines the importance of longitudinal studies.

Although 84% of the children with cancer were treated with intravenous ampicillin for a median of 29 days per year during the study period, the occurrence of ampicillin resistance was not significantly higher in the cancer patients as compared to the healthy controls at the end of the study period. This may indicate that aminopenicillin administered intravenously has only a modest effect on selection of intestinal resistance and possibly less impact than oral administration, which was the main route of administration of aminopenicillin to children with CF (Fig 1, S2 Table). This is supported by Zhang et al. who demonstrated a much greater increase of faecal, resistant bacterial populations in mice after oral ampicillin administration compared to intravenous administration [50]. Ampicillin is mainly excreted by the kidneys, and biliary excretion is of minor importance [51]. Thus, the ampicillin exposure of the gut flora during parenteral administration seems far less than with per oral treatment. Some older studies have shown increased prevalence of faecal ampicillin-resistant enterobacteria associated with intravenous administration of ampicillin in neonates [52, 53]. However, the studies of the effect of aminopenicillin treatment on the gut flora have almost exclusively investigated peroral drug administration [8]. Clinical studies comparing the impact of intravenous versus peroral administration of antibiotics on the gut flora are lacking. The present study was not designed to evaluate the effect of intravenous versus peroral administration of antibiotics on resistance in the faecal flora. Also, a comparison of the effect of antibiotic treatment in children with CF (mainly oral) and children with cancer (mainly intravenous) on antibiotic resistance in faecal bacteria could not be performed since these two groups represent very different diseases and antibiotic treatment strategies, including spectre of antibiotics used and duration of treatment. Repeated courses of antibiotics given to the children with CF from a young age probably have an impact on the higher prevalence of faecal carriage of resistant enterobacteria that was found in these children in our study.

Tetracycline-resistant *E*. *coli* and other *Enterobacteriaceae* were detected in all three study groups (Table 3). Treatment with tetracycline is contraindicated in children below the age of eight years due to the risk of discolouration of the teeth and effects on bone growth, and no child in our study had ever been treated with tetracyclines. The high level of tetracycline resistance without any apparent selection pressure from tetracycline exposure is indicative of transfer of tetracycline-resistant strains and/or genetic elements from environmental sources. Several studies have documented faecal carriage of tetracycline-resistant strains in children, including infants [22, 54].

The prevalence of resistance to third-generation cephalosporins was not higher in the cancer group (in whom 66% of the children were treated for a median of 31 days per calendar year with this class of antibiotics) as compared to the untreated healthy children. de Man et al. detected a high risk of colonisation with enterobacteria resistant to cefotaxime in neonates treated with intravenous amoxicillin plus cefotaxime [48]. Prevot et al. found that intestinal colonization with cefotaxime-resistant *Enterobacteriaceae* in oncological patients was strongly associated with individual exposure to cefotaxime [55]. Except for this, few studies have actually documented a correlation between third-generation cephalosporin treatment and faecal carriage of resistance to these agents in individuals. The difference in the prevalence of third-generation cephalosporin-resistant *E*. *coli* between the CF group and the control group barely reached statistical significance in the last faecal sample (Table 3). The overall number of resistant isolates was however low, and *E*. *coli* isolates carrying acquired ESBL genes were detected in samples from only one single CF patient and from two cancer patients. The low level of third-generation cephalosporin-resistance in *E*. *coli* as well as in other *Enterobacteriaceae* in our study corresponds well with surveillance program reports from Norway during the same time period [23]. We speculate that low resistance rates in the community may be of importance for the low level of selection of third-generation cephalosporin-resistant strains observed in our two patient groups treated with these agents.

The prevalence of gentamicin resistance was low in all the study groups, including the group of cancer patients in whom 84% of the children had been treated with aminoglycosides for a median of 33.5 days per year in the study period. Aminoglycosides are primarily excreted by the kidneys and less than 1% is eliminated in the faeces [56]. This may explain why no effect of aminoglycoside treatment on enterobacterial resistance was detected in our study.

Samples growing *Klebsiella* spp. and some of the other non-*E*. *coli* enterobacterial species were significantly more prevalent in the CF group compared to controls (Table 2). These species are intrinsically resistant to some antibiotics, including ampicillin [57], and thus have a selective advantage and may emerge during amoxicillin treatment.

Analyses of WGS data detected the presence of resistance determinants that explained the observed phenotypic resistance in all 28 *E*. *coli* isolates examined in this study. This included mutations (-42:C->T, -18:G->A) in the promoter region of the chromosomal *ampC* gene in two isolates resistant to third-generation cephalosporins. The -42:C->T nucleotide change has been shown to increase *ampC* expression 22-fold compared to wild-type *E*. *coli* [37]. Two previously described *gyrA* mutations and one or two *parC* mutations were detected in all the ciprofloxacin-resistant isolates (Table 6 and S4 Table); in accordance with other studies that reported that more than one *gyrA* mutation confer phenotypic ciprofloxacin resistance [40, 58].

The MLST-analyses showed no clustering of isolates that could indicate patient-to-patient transfer or an outbreak. The observation of *E*. *coli* isolates of different STs in the same patient, all harbouring *bla*_CTX-M-1_, indicates transfer of a mobile genetic element carrying this resistance gene between *E*. *coli* strains in the gut of the individual patient. This within-host diversity has previously been shown for ESBL-producing *E*. *coli* [59, 60].

Our study has some limitations. The children with cancer comprised a very heterogeneous group of malignant diseases that vary in treatment intensity and duration. This probably influences the risk of acquiring infections and the level of antibiotic exposure within this study group. The number of individuals within each of the different malignancies was too small to perform subgroup analyses of antibiotic exposure and resistance. Further, we cannot exclude that repeated exposures to cytostatic chemotherapy per se also have an impact on the gut flora, including the level of resistance. Studies on this issue are lacking, although some researches have studied the effects of antineoplastic drugs, alone or in combination with antibiotics, on bacterial growth *in vitro*, with conflicting results [61–64].

The faecal samples in this study were collected several years ago, and an obvious limitation of our study is lack of data from the present time period. However, the low level of resistance in Norway at the time of the study [23], allowed us to evaluate the impact of the antibiotic treatment with minor influence of influx of resistance from the surroundings. Nevertheless, a replication of such a study in Norway at present would be of great interest. Another limitation of the study is the comparatively small number of children with CF and cancer included, both being relatively rare diseases. Thus, the lack of statistically significant correlations between antibiotic treatment and occurrence of antibiotic resistance in our study may be due to too low power to study the effect of exposure to each individual antibiotic within each patient group.

## Conclusions

In this study we found that the prevalence of faecal carriage of enterobacteria resistant to ampicillin and trimethoprim-sulfamethoxazole was significantly higher in children with CF as compared to healthy children. A likely explanation is a selection pressure from treatment with large amounts of these antibiotics in the CF patients. However, the prevalence of resistant faecal enterobacteria was not higher in children with cancer after repeated courses of mainly intravenous antibiotic treatment compared to healthy children with no or a very low level of antibiotic exposure. The level of enterobacterial resistance to third-generation cephalopsorins, ciprofloxacin and gentamicin was low in all three study groups. We speculate whether the mode of administration of antibiotics and the level of antimicrobial resistance in the community may have an impact on emergence of resistance in intestinal enterobacteria during antibiotic treatment. Further studies comparing the effect of intravenous versus peroral administration of antibiotics on the gut flora are needed to assess such a hypothesis. WGS analyses detected acquired resistance genes and/or chromosomal mutations that explained the observed phenotypic resistance in 28 examined multidrug-resistant *E*. *coli* isolates.

## Supporting information

S1 Data Epidemiology and faecal sample results. (SAV)

S1 Table Assembly data from 28 whole genome sequenced *E*. *coli* isolates. (XLSX)

S2 Table Antibiotic treatment in 31 children with cystic fibrosis and 44 children with cancer. (DOCX)

S3 Table Antibiotic treatment among 33 of the 70 healthy controls. (XLSX)

S4 Table Sequence type, phenotypic resistance patterns and corresponding genetic resistance determinants in 28 *Escherichia coli* isolates from faecal samples from children with cystic fibrosis and cancer and healthy controls. (XLSX)

S5 Table Molecular investigation of *Enterobacteriaceae* isolates (non-*E*. *coli*) with reduced susceptibility to third-generation cephalosporins. (DOCX)
