# Correction: Impact of extensive antibiotic treatment on faecal carriage of antibiotic-resistant enterobacteria in children in a low resistance prevalence setting

**DOI:** 10.1371/journal.pone.0193439

**Published:** 2018-02-21

**Authors:** Per Kristian Knudsen, Petter Brandtzaeg, E. Arne Høiby, Jon Bohlin, Ørjan Samuelsen, Martin Steinbakk, Tore G. Abrahamsen, Fredrik Müller, Karianne Wiger Gammelsrud

## Notice of Republication

This article was republished on November 10, 2017 to replace incorrect versions of S2 Table and S3 Table. Please download this article again to view the corrected tables.
