# Supplementary material for: Impact of extensive antibiotic treatment on faecal carriage of antibiotic-resistant enterobacteria in children in a low resistance prevalence setting
Source: PLoS One. 2017 Nov 7;12(11):e0187618. doi: 10.1371/journal.pone.0187618 (PMC5675440; doi:10.1371/journal.pone.0187618)
Supplement: S2 Table — (DOCX) [file pone.0187618.s003.docx]

**S2 Table. Antibiotic treatment in 31 children with cystic fibrosis (CF) and 44 children with cancer^a^.**

| Antibiotic treatment | CF (n=31) | Cancer (n=44) | p-values^b^ |
| --- | --- | --- | --- |
| Phenoxymethylpenicillin |  |  |  |
| Number of patients (%) | 6 (19.4) | 9 (20.5) | 0.91 |
| Number of days^c^ | 23.5 (12.5-32.8) | 8.5 (5.9-12.7) | 0.02 |
| Amoxicillin^d^ |  |  |  |
| Number of patients (%) | 17 (54.8) | 6 (13.6) | <0.001 |
| Number of days^c^ | 18.9 (5.7-52.3) | 8.4 (5.5-10.7) | <0.001 |
| Ampicillin^e^ |  |  |  |
| Number of patients (%) | 1 (3.2) | 37 (84.1) | <0.001 |
| Number of days^c^ | 20.0 | 28.9 (2.8-78.7) | ND |
| Cloxacillin or dicloxacillin |  |  |  |
| Number of patients (%) | 13 (41.9%) | 13 (29.5%) | 0.27 |
| Number of days^c^ | 35.6 (6.2-88.4) | 13.8 (2.0-39.1) | 0.003 |
| 1. or 2. generation cephalosporins^f^ |  |  |  |
| Number of patients (%) | 18 (58.1%) | 18 (40.9%) | 0.14 |
| Number of days^c^ | 28.0 (8.1-69.4) | 9.6 (3.0-33.2) | 0.001 |
| 3. generation cephalosporins^e,g^ |  |  |  |
| Number of patients (%) | 8 (25.8) | 29 (65.9) | 0.001 |
| Number of days^c^ | 17.9 (7.6-21.5) | 31.1 (3.9-89.1) | <0.001 |
| Meropenem |  |  |  |
| Number of patients (%) | 2 (6.5%) | 7 (15.9%) | 0.22 |
| Number of days^c^ | 14.3 (8.4-20.3) | 11.0 (5.9-25.9) | ND |
| Aminoglycosides^e,h^ |  |  |  |
| Number of patients (%) | 9 (29.0) | 37 (84.1) | <0.001 |
| Number of days^c^ | 18.7 (7.6-40.6) | 33.5 (3.6-85.3) | 0.005 |
| Ciprofloxacin^d^ |  |  |  |
| Number of patients (%) | 7 (22.6) | 0 | 0.001 |
| Number of days^c^ | 40.1 (7.3-57.2) | 0 | ND |
| Trimethoprim-sulfamethoxazole |  |  |  |
| Number of patients (%) |  |  |  |
| Treatment^i^ | 20 (64.5%) | 13 (29.5) | 0.003 |
| Prophylaxis | 0 | 31 (70.5) | <0.001 |
| Treatment and/or prophylaxis | 20 (64.5) | 37 (84.1) | 0.05 |
| Number of days^c,j^ | 59.8 (7.9-91.6) | 28.4 (4.0-239.8) | 0.43 |
| Macrolides^k^ |  |  |  |
| Number of patients (%) | 10 (32.3) | 5 (11.4) | 0.03 |
| Number of days^c^ | 21.4 (7.6-156.7) | 9.4 (3.0-10.4) | 0.04 |
| Clindamycin |  |  |  |
| Number of patients (%) | 8 (25.8) | 2 (4.5) | 0.008 |
| Number of days^c^ | 17.4 (7.6-21.6) | 27.3 (24.2-30.4) | ND |
| Vancomycin |  |  |  |
| Number of patients (%) | 0 | 19 (43.2) | <0.001 |
| Number of days^c^ | 0 | 24.3 (6.0-62.9) | ND |
| Metronidazole |  |  |  |
| Number of patients (%) | 0 | 23 (52.3) | <0.001 |
| Number of days^c^ | 0 | 27.1 (3.8-220.3) | ND |

^a^ For one of the 32 CF patients and for one of the 45 cancer patients included in the study no antibiotic treatment was registered since a faecal sample was provided only at the time of inclusion into the study

^b^ Some p-values were not determined (ND) due to small numbers

^c^ Median (range) number of days per calendar year with antibiotic treatment in patients who received at least one course of treatment during the study period

^d^ Oral treatment only

^e^ Intravenous treatment only

^f^ Mainly oral cephalexin in the CF group, mainly intravenous cefuroxim in the cancer group

^g^ Ceftazidime in the CF group. Cefotaxime, ceftazidime and/or ceftriaxone in the cancer group

^h^ Mainly tobramycin in the CF group, mainly gentamicin in the cancer group. Inhaled tobramycin not included

^i^ Only oral treatment in the CF group, mainly oral treatment in the cancer group

^j^ Prophylaxis not included

^k^ Including two CF patients that received azitromycin on a regular basis (three days per week)
