# Supplementary material for: Impact of extensive antibiotic treatment on faecal carriage of antibiotic-resistant enterobacteria in children in a low resistance prevalence setting
Source: PLoS One. 2017 Nov 7;12(11):e0187618. doi: 10.1371/journal.pone.0187618 (PMC5675440; doi:10.1371/journal.pone.0187618)
Supplement: S5 Table — (DOCX) [file pone.0187618.s006.docx]

**S5 Table. Molecular investigation of *Enterobacteriaceae* isolates (non-*E. coli*) with reduced susceptibility to third-generation cephalosporins.**

| **SPECIES** | **Subject^1^** | **Phenotypic resistance^2^** | **ESBLA phenotypic tests^3^** | **AmpC phenotypic tests^4^** | **Conclusion** | Note |
| --- | --- | --- | --- | --- | --- | --- |
| *Citrobacter freundii* | **CP (2)** | CAZ, CTX | Neg./inconclusive | Positive | Cromosomal AmpC | *C. freundii* contains intrinsic cromosomal AmpC (CMY or CFE) |
| *Citrobacter freundii* | **HC (2)** | CAZ, CTX | Neg./inconclusive | Positive | Cromosomal AmpC | *C. freundii* contains intrinsic cromosomal AmpC (CMY or CFE) |
| *Citrobacter koseri/sedlakii* | **CP (2)** | CAZ | Neg./inconclusive | Negative | Negative ESBL_A_/AmpC - most likely wild type |  |
| *Enterobacter asburiae* | **CP (1)** | CAZ, CTX | Neg./inconclusive | Positive | Derepressed cromosomal AmpC |  |
| *Enterobacter asburiae* | **HC (1)** | CAZ, CTX | Neg./inconclusive | Positive | Derepressed cromosomal AmpC |  |
| *Enterobacter cloacae* | **CP (1)** | CAZ, CTX | Neg./inconclusive | Positive | Derepressed cromosomal AmpC |  |
| *Enterobacter cloacae* | **HC (2)** | CAZ, CTX | Neg./inconclusive | Positive | Derepressed cromosomal AmpC |  |
| *Enterobacter cloacae* | **HC (2)** | CAZ, CTX | Neg./inconclusive | Positive | Derepressed cromosomal AmpC |  |
| *Enterobacter cloacae* | **HC (2)** | CAZ, CTX | Neg./inconclusive | Positive | Derepressed cromosomal AmpC |  |
| *Hafnia alvei* | **HC (1)** | CAZ, CTX | Neg./inconclusive | Positive | Derepr. cAmpC + imperm/effluks | *H. alvei* contains intrinsic cromosomal AmpC (ACC-type) |
| *Hafnia alvei* | **CP (2)** | CAZ, CTX | Neg./inconclusive | Positive | cAmpC | *H. alvei* contains intrinsic cromosomal AmpC (ACC-type) |
| *Hafnia alvei* | **CP (2)** | CAZ, CTX | Neg./inconclusive | Positive | cAmpC |  |
| *Morganella morganii* | **HC (1)** | CAZ, CTX | Neg./inconclusive | Positive | cAmpC | *M. morganii* contains intrinsic cromosomal AmpC (DHA-type) |

^1^ Five cancer patients (CP) and six healthy controls (HC), sample 1 or 2.

^2^ Ceftazidime; CAZ, cefotaxime; CTX

^3^ MIC gradient strips and BD ESBL combination discs

^4^ MIC gradient strips and AmpC Confirm ID kit ROSCO
